# Clinical features of central nervous system infections and experience in differential diagnosis from neuropsychiatric lupus erythematosus in a cohort of 8491 patients with systemic lupus erythematosus

**DOI:** 10.1186/s13075-019-1971-2

**Published:** 2019-08-19

**Authors:** Mengdi Jiang, Xiaochun Shi, Xin Gao, Jingwen Niu, Xiaomin Hu, Lidan Zhao, Xuan Zhang

**Affiliations:** 1 Department of Rheumatology and Clinical Immunology, Peking Union Medical College Hospital, Clinical Immunology Center, Chinese Academy of Medical Sciences and Peking Union Medical College, The Ministry of Education Key Laboratory, Beijing, China; 2 Department of Infectious Disease, Peking Union Medical College Hospital, Chinese Academy of Medical Sciences and Peking Union Medical College, Beijing, China; 3 Department of Radiology, Peking Union Medical College Hospital, Chinese Academy of Medical Sciences and Peking Union Medical College, Beijing, China; 4 Department of Neurology, Peking Union Medical College Hospital, Chinese Academy of Medical Sciences and Peking Union Medical College, Beijing, China; 5 Clinical Immunology Center, Peking Union Medical College Hospital, Chinese Academy of Medical Sciences and Peking Union Medical College, Beijing, China

**Keywords:** Central nervous system, Infection, Systemic lupus erythematosus, Risk factor, Scoring system

## Abstract

**Background:**

In clinical practice, discrimination between central nervous system (CNS) infections in patients with systemic lupus erythematosus (SLE) and neuropsychiatric lupus erythematosus (NPSLE) could be urgent and critical yet extremely challenging. Given this, this study aimed to investigate the clinical features and outcomes of infections in the CNS in patients with SLE and to establish a simplified scoring system for guiding the discrimination of CNS infections from NPSLE.

**Methods:**

A total of 95 patients who were identified as having CNS infections among 8491 SLE patients between January 1992 and January 2018 were included in this retrospective study. NPSLE patients admitted at the same period were randomly selected for comparison. Key factors either clinically valuable or statistically significant for discriminating CNS infections from NPSLE were integrated to build a simplified scoring system. Another group of 22 SLE patients complicated with suspected newly onset of CNS infections or NPSLE admitted after January 2018 was enrolled to verify the utility of the scoring system.

**Results:**

Sixty-three positive pathogens were identified in 59 patients of the total 95 CNS infection cases. Compared with the NPSLE group, the CNS infections group had a longer disease duration (21.0 [3.0–50.0] vs. 1.0 [0–22.0] months, *P* < 0.05), exhibited more fever (96.8% vs. 23.2%, *P* < 0.001) and polymorphonuclear leukocyte leukocytosis in the cerebrospinal fluid (CSF) (45.6% vs. 0.5%, *P* < 0.05), and had significantly decreased CSF glucose (2.0 ± 1.3 vs. 3.3 ± 0.9 mmol/L, *P* < 0.01), whereas hypocomplementemia seemed to be a strong hint of NPSLE (44.6% vs. 77.4%, *P* < 0.001). A simplified scoring system integrated with 8 key factors was established for guiding clinical differential diagnosis. By setting the cutoff value at 4 and verifying in a group of SLE patients complicated with newly occurred suspected CNS infection or NPSLE, a sensitivity of 85.7% and specificity of 93.3% with the area under the curve (AUC) being 0.93 (95%CI 0.80–1.00) were obtained.

**Conclusions:**

CNS infections are a fatal complication of SLE and can be difficult to discriminate from NPSLE. A simplified scoring system may help to make preliminary discrimination of CNS infections from NPSLE.

**Electronic supplementary material:**

The online version of this article (10.1186/s13075-019-1971-2) contains supplementary material, which is available to authorized users.

## Background

Systemic lupus erythematosus (SLE) is a typical autoimmune disorder characterized by widespread immune deregulation, resulting in systemic inflammation and multi-organ impairments. Immunosuppressive agents, including glucocorticoids (GCs) and disease-modifying antirheumatic drugs (DMARDs), are the main therapeutic tools but may increase the risk of severe infections [1–4]. Infections are one of the major causes of morbidity and mortality in SLE patients. According to previous studies, infections are estimated to be responsible for 11 to 50% of deaths in SLE [5–8]. In clinical practice, discrimination between infections and SLE flares could be extremely challenging, for example, infections may mimic symptoms of SLE, leading to confusion over the diagnosis and delay of treatment. Central nervous system (CNS) infections constitute up to 3% of all infections in lupus patients, and although not very common, these infections are life-threatening and severely disabling [9]. More importantly, CNS infections in SLE patients are easily confused with neuropsychiatric lupus erythematosus (NPSLE) which is a challenging complication of SLE that requires vigorous treatment with high-dose GCs and DMARDs. These two conditions may exhibit similar symptoms and phenotypes, yet they require completely different therapeutic strategies. CNS infections usually have a high mortality rate unless an accurate diagnosis is promptly made and the appropriate therapeutic intervention is initiated at the very early stage.

To better understand and manage CNS infections in patients with SLE, we conducted this study in 8491 SLE in-patients admitted in the past 26 years to Peking Union Medical College Hospital (PUMCH), which is the tertiary referral center in China. In this study, we aim to not only identify the clinical features and etiology of CNS infections, but also provide the diagnostic clues for discriminating CNS infections from NPSLE among SLE patients. To our knowledge, this is the largest cohort study in this field to date and may provide practical guidance for clinicians to distinguish these two severe and confusing complications.

## Methods

### Patient selection

PUMCH in-patient register database system was used for two-step screening electronically. First, we screened 8491 in-patients with a final diagnosis of “systemic lupus erythematosus” who were consecutively admitted to PUMCH from January 1992 to January 2018. Then, to identify probable cases of CNS infections, we searched the patient records for keywords including the following: “central nervous system infection,” “intracranial infection,” “encephalitis,” “meningitis,” “meningoencephalitis,” and “brain abscess.” To identify probable cases of NPSLE, we searched the patient records for keywords including the following: “lupus encephalopathy” and “neuropsychiatric lupus.” Patients with NPSLE would constitute the pool of candidates for the control group. Ninety-five SLE patients were confirmed to have CNS infections according to the criteria mentioned below. An equal number of NPSLE patients were randomly selected, using a computational algorithm that matched the age and gender. Medical records of these cases were reviewed, and those lacked key information were removed from the study. Relevant information was collected, and uncertain cases were discussed by a multiple disciplinary team (MDT) consisting of two rheumatologists, one specialist of infectious diseases, one radiologist, and one neurologist. If the MDT could not come to a consensus on a particular case, that case would be excluded from the study. If a patient in the NPSLE group had a concurrent infection, they were also excluded from the study. Further details of the screening process are shown in Additional file 1. All patients fulfilled the 1982 American College of Rheumatology (ACR) classification criteria for SLE. NPSLE refers to the neurologic and psychiatric syndromes involving CNS categorized by ACR subcommittee in 1999 [10] with excluding causes other than lupus. The definitive diagnosis of CNS infections was based on (1) clear etiological evidence, including positive finding of the pathogens from cerebrospinal fluid (CSF) or brain lesion biopsy via microorganism culture or smear with gram, acid-fast, or India ink staining; (2) indirect etiological evidence, including positivity in pathogen antigen/antibody detection, such as the cryptococcal antigen latex agglutination system (CALAS) test, and Cysticercus cellulosae antibody detection, or positivity for pathogen DNA detected by polymerase chain reaction (PCR); (3) clinical diagnosis confirmed by expert opinions based on comprehensive evaluation of clinical manifestations, CNS examinations, laboratory findings, and typical neuroimaging results strongly suggestive of CNS infections. This study was approved by the Ethics Committee of PUMCH (S-K 807). Since this retrospective study was based on reviewing the medical records obtained for clinical purpose, the requirement of informed consent was waived and general confidential principles were obeyed.

### Study design

#### Demographics, clinical characteristics, and outcomes in SLE patients with CNS infection

The medical records of these patients were systematically reviewed and evaluated by the MDT, and all clinical data was collected. Follow-up clinical evaluations were conducted in all available patients, to update the case outcomes. Demographic features, medical history, clinical manifestations, laboratory findings, previous treatments history including the use of GCs and DMARDs, and systemic lupus erythematosus disease activity index 2000 (SLEDAI-2K) at the onset of CNS infection or NPSLE were recorded and presented in Table 1. All these patients underwent CSF examinations, including white blood cell (WBC) count and classification, and the protein, glucose, and chloride levels measurement. The etiological tests included microorganism culture; smear with gram, acid-fast, and India ink staining; CALAS test; and *Mycobacterium tuberculosis* DNA detection by PCR. Antibodies against viruses and parasites, such as Cysticercus cellulosae, were detected in cases as needed. Neuroimaging, such as magnetic resonance imaging (MRI), was conducted for all cases without contraindications. Otherwise, computed tomography (CT) scans were performed.

**Table 1:** Baseline demographics, clinical features and treatments in SLE with infections vs. NPSLE

Items	CNS infections (*n* = 95)	NPSLE (*n* = 95)	*P* value
Sex, female, *n* (%)	81 (85.3)	81 (85.3)	1.000
Age at SLE diagnosis, year, mean (SD)	31.0 (13.9)	30.8 (14.1)	0.897
Age at onset^§^, years, mean (SD)	34.6 (13.7)	32.3 (14.7)	0.276
SLE disease duration^&^, months, median (IQR)	21.0 (3.0–50.0)	1.0 (0–22.0)	**< 0.001**
System involvement of SLE, *n* (%)			
Lupus nephritis	71 (74.7)	69 (72.6)	0.742
NPSLE	26 (27.4)	95 (100)	**< 0.001**
Hematological	65 (68.4)	62 (65.3)	0.644
Mucocutaneous	79 (83.2)	66 (69.5)	**< 0.05**
Musculoskeletal	51 (53.7)	52 (54.7)	0.884
Cardiovascular	12 (12.6)	22 (23.2)	0.058
Pulmonary	6 (6.3)	18 (18.9)	**< 0.05**
Medical history*, *n* (%)			
Pulmonary tuberculosis	5 (5.3)	5 (5.3)	1.000
Fungal infections	2 (2.1)	1 (1.1)	1.000
Diabetes mellitus	9 (9.5)	4 (4.2)	0.151
Herpes zoster infections	9 (9.5)	1 (1.1)	**< 0.01**
Previous treatment*			
Pulse GCs, *n* (%)	35 (36.8)	7 (7.4)	**< 0.001**
Average daily prednisone dose (or equivalent) in recent 6 months, mg/day, mean (SD)	43.5 (44.2)	21.8 (37.5)	**< 0.001**
DMARDs in recent 6 months, *n* (%)	67 (70.5)	36 (37.9)	**< 0.001**
CTX/MMF in recent 1 year, *n* (%)	49 (51.6)	17 (17.9)	**< 0.001**
Neuropsychiatric symptoms^§^, *n* (%)			
Fever	92 (96.8)	22 (23.2)	**< 0.001**
Headache	85 (89.5)	42 (44.2)	**< 0.001**
Seizure	24 (25.3)	35 (36.8)	0.085
Psychosis	17 (17.9)	31 (32.6)	**< 0.05**
Cognitive dysfunction	17 (17.9)	32 (33.7)	**< 0.05**
Acute confusional state	49 (51.6)	19 (20.0)	**< 0.001**
Anxiety disorder	2 (2.1)	10 (10.5)	**< 0.05**
CSF examination^§^			
Pressure ≥ 300 mmH_2_O, *n* (%)	47 (51.1)	9 (9.5)	**< 0.001**
WBCs, 10^6^/L, mean (SD)	635 (1470)	3 (12)	**< 0.001**
PMN ratio, %, mean (SD)	45.6 (36.0)	0.5 (2.4)	**< 0.001**
Protein, g/L, mean (SD)	2.13 (3.78)	0.64 (0.65)	**< 0.001**
Glucose, mmol/L, mean (SD)	2.0 (1.3)	3.3 (0.9)	**< 0.001**
Laboratory blood test at onset^§^			
WBCs, 10^6^/L, mean (SD)	9084 (5898)	6497 (3508)	**< 0.001**
PMN ratio, %, mean (SD)	82.2 (10.3)	75.8 (11.9)	**< 0.001**
Lymphocytes, 10^6^/L, mean (SD)	923 (771)	1032 (758)	0.354
Hypocomplementemia, *n* (%)	41 (44.6)	72 (77.4)	**< 0.001**
IgG, g/L, mean (SD)	13.6 (7.6)	12.2 (7.4)	0.236
ESR, mm/h, mean (SD)	54.8 (39.5)	48.2 (31.8)	0.215
SLEDAI-2K score, mean (SD)	7.5 (7.3)	18.4 (5.6)	**< 0.001**
SLICC/ACR Damage Index, mean (SD)	1.03 (1.04)	1.01 (0.88)	0.151
Morality rate^#^, *n* (%)	26 (27.4)	13 (13.7)	**< 0.05**

Pulse GCs are defined as equal to or greater than 500 mg/day methylprednisolone infusion for consecutive 3~5 days; DMARDs, including cyclophosphamide, mycophenolate mofetil, methotrexate, cyclosporin, tacrolimus, azathioprine, hydroxychloroquine, leflunomide

*CNS* central nervous system, *GCs* glucocorticoids, *NPSLE* neuropsychiatric lupus erythematosus, *DMARDs* disease-modifying antirheumatic drugs, *CTX* cyclophosphamide, *MMF* mycophenolate mofetil, *CSF* cerebrospinal fluid, *ESR* erythrocyte sedimentation rate, *WBCs* white blood cells, *PMN* polymorphonuclear leukocyte, *IgG* immunoglobulin G, *SLEDAI-2K* systemic lupus erythematosus disease activity index 2000. *SLICC/ACR* Systemic Lupus International Collaborating Clinics/American College of Rheumatology

^§^Evaluated within 2 weeks of CNS infection or NPSLE onset

^&^Disease duration from SLE diagnosis to CNS infections or NPSLE onset

*Evaluated history before the diagnosis of CNS infection or NPSLE

^#^Evaluated within 1 year of diagnosis of CNS infection or NPSLE

Significant *p* values are shown in bold typeface

#### A simplified scoring system for discriminating CNS infections from NPSLE in SLE patients

A simplified scoring system comprising 8 items was established for guiding clinical practice. Seventy-five out of 95 cases (79%) among the CNS infections group and the NPSLE group were integrated to determine the risk factors. Four factors were concluded from a univariate analysis and fixed by further multivariate logistic stepwise regression with the cutoff values decided by the receiver operating characteristic (ROC) curve. The MDT then voted on the other items, and four criteria for the diagnosis of CNS infection vs. NPSLE, based on extensive clinical experience, were selected and cutoff values determined.

#### Scoring system verification

The scoring system was verified in the remaining 20 cases (21%). Sensitivity and specificity were calculated, and the scoring system was optimized. The scoring system was then applied to 22 SLE patients who were admitted to PUMCH after January 2018, with a recent onset of neuropsychiatric symptoms, yet uncertain diagnosis of CNS infection or NPSLE. The final clinical diagnosis was traced and then compared to the predicted diagnosis based on our scoring model. Positive and negative predictive values were calculated for accuracy evaluation of prejudgement.

### Statistical analysis

The chi-square (*χ*^2^ test), Student *t*, and Mann-Whitney tests were used to compare the categorical data, numerical data with a normal distribution, and numerical data without a normal distribution, respectively. Data with a normal distribution are displayed with the mean and standard deviation (SD). Non-parametrially distributed data are represented as the median (interquartile range [IQR]). A univariate analysis was performed to determine the variables associated with CNS infections compared with NPSLE and factors associated with mortality. A multivariate logistic stepwise regression was performed with variables with *P* value less than 0.05 in univariate analysis, a stepwise forward method used likelihood ratio test (LR) was used for variables entering the model. The ROC curve was utilized to find the cutoff values of variables with statistical significance for optimal event discrimination. Survival analysis was performed to compare the prognosis of the two groups using the Kaplan-Meier method calculated by log-rank test. The Statistical Package for the Social Sciences 20.0 (SPSS, Inc., Chicago, IL, USA) was used for all data analysis. A *P* value less than 0.05 was considered statistically significant.

## Results

### Demographic, clinical features and etiological detection of SLE patients complicated with CNS infections

Of the 8491 SLE in-patients in PUMCH, 95 patients (1.12%) were identified as having CNS infections, which is similar to what has been previously reported in the literature of 0.54–2.25% [11–13]. The female to male ratio of this cohort was 5.8:1 (81 vs. 14). The mean age at the time of SLE diagnosis was 31.0 ± 13.9 years, while the mean age at the onset of CNS infections was 34.6 ± 13.7 years. The median lupus disease duration prior to the onset of neuropsychiatric symptom in the CNS infections group was significantly longer than that in the NPSLE group (median 21.0 vs. 1.0 months, IQR 3–50 vs. 0–22 months, *P* < 0.001). The median time interval from the onset of neuropsychiatric symptom to the diagnosis of CNS infections was 9.0 days (IQR 2.0–36.0 days) (Table 1).

Regarding previous treatments, 36.8% of patients in the CNS infections group had been treated with pulse GCs, in contrast to 7.4% of patients in the NPSLE group (*P* < 0.001). The daily dose of prednisone or equivalent in the past 6 months in the CNS infections group was much higher than that in the NPSLE group (43.5 ± 44.2 mg vs. 21.8 ± 37.5 mg, *P* < 0.001). Sixty-seven (70.5%) patients in the CNS infections group had received at least one DMARD (including cyclophosphamide (CTX), mycophenolate mofetil (MMF), methotrexate, cyclosporin, tacrolimus, azathioprine, hydroxychloroquine, and leflunomide) in the past 6 months, in contrast to 36 (37.9%) patients in the NPSLE group (*P* < 0.001) (Table 1).

For the CNS infections group, the most common clinical manifestations at the onset of infection were fever (96.8%), headache (89.5%), and acute confusional state (51.6%), all of which occurred more frequently than those in the NPSLE group (23.2%, 44.2%, 20.0%, respectively, all *P* < 0.001) (Table 1). Lumbar puncture revealed that the CNS infections group had more severe intracranial hypertension, with 80 patients (84.8%) having increased intracranial pressure (ICP) (> 180 mmH_2_O, normal range 80–180 mmH_2_O) and 47 (51.1%) having extremely high ICP (> 300 mmH_2_O), compared to 50.5% and 9.5%, respectively, in the NPSLE group (both *P* < 0.001). The median WBC count in the CSF and the proportion of polymorphonuclear (PMN) leukocyte were both expected higher in the CNS infections group than those in the NPSLE group (635 ± 1470/mm^3^ vs. 3 ± 12/mm^3^ and 45.6 ± 36.0% vs. 0.5 ± 2.4%, respectively, *P* < 0.05). Also, the CSF protein levels were elevated (2.13 ± 3.78 vs. 0.64 ± 0.65 g/L, *P* < 0.001) remarkably, and the glucose level decreased (2.0 ± 1.3 vs. 3.3 ± 0.9 mmol/L, *P* < 0.001) significantly in the CNS infections group than in the NPSLE group (Table 1).

**Fig. 1:**
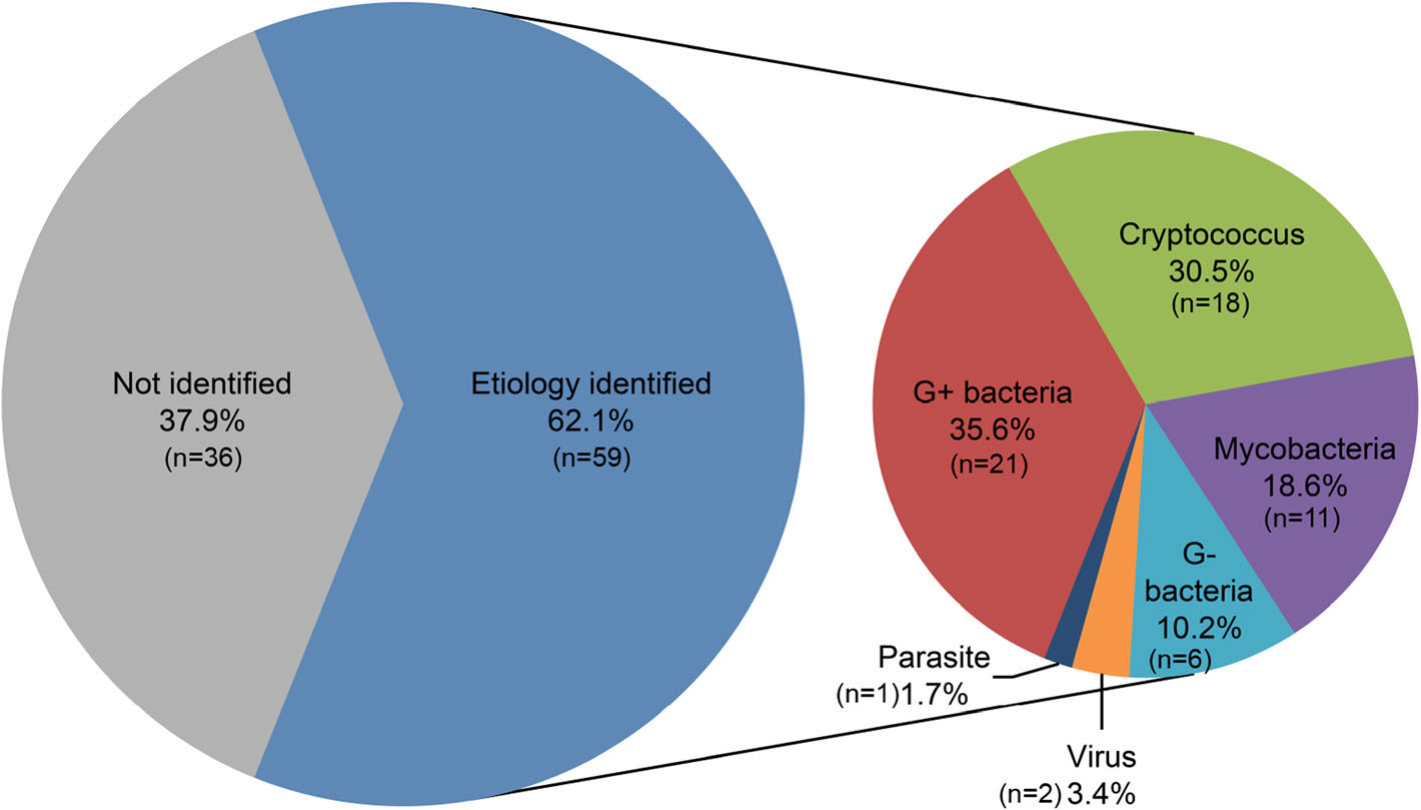
The etiology of 59 SLE patients with CNS infections. The G+ bacteria included *Listeria monocytogenes, Nocardia asteroides, Staphylococci epidermidis, Staphylococci hominis, Steptococcus pneumonia, Enterococcus faecium, Corynebacterium diphtheria* and species undefined; G- bacteria included *Pseudomonas aeruginosa, Acinetobacter lwoffii, Acinetobacter Baumannii* and species undefined; Virus included *Epstein-Barr virus* and *Herpes simplex virus*; Parasite referred *Cysticercus botryoides*. Species undefined, positive result of smear with gram staining but negative of microorganism culture; G+ bacteria, Gram-staining-positive bacteria; G- bacteria, Gram-staining-negative bacteria

Etiological tests identified 63 well-defined pathogens in 59 patients (62.1%), with 4 patients having been detected 2 pathogens at the same time. Common *Bacteria* were identified in 27 patients (45.8%), followed by *Cryptococcus* in18 patients (30.5%) and mycobacteria in 11 patients (18.6%). Among the bacterial infections, the top 3 species were *Listeria monocytogenes* in 10 patients (16.9%), *Nocardia asteroides* in 4 patients (6.8%), and staphylococci in 3 patients (5.1%). Two patients (3.4%) were identified as having viral meningoencephalitis: one was confirmed to have Epstein-Barr virus (EBV) meningoencephalitis with the evidence of high copies of EBV DNA in the CSF; the other was found to have high titers of anti-herpes simplex virus-IgM in the CSF. One patient (1.7%) was diagnosed with cerebral cysticercosis, based on the positive anti-Cysticercus cellulosae antibody both in CSF and serum. Detailed pathogenic information for these patients was shown in Fig. 1.

**Fig. 2:**
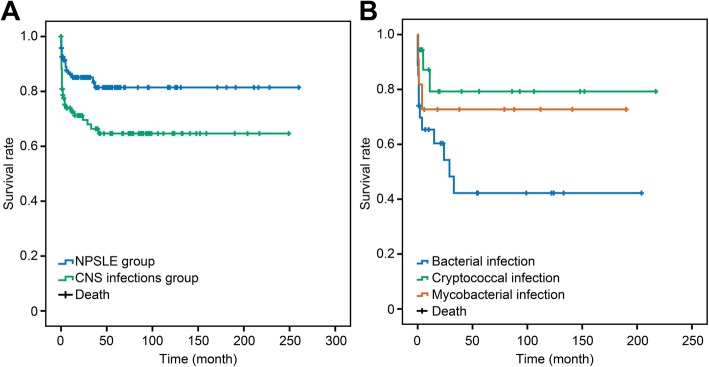
Survival rates of SLE with CNS infections vs. NPSLE and etiology of CNS infections. **a** Survival rates of SLE patients with CNS infections and NPSLE. The survival rate of the CNS infections group was significantly lower than that of the NPSLE group (*P* < 0.01). **b** Survival rates of CNS infections in SLE patients with three different types of etiology. The survival rate of the bacterial subgroup was significantly lower than that of the other two subgroups, but the difference among the three groups was not statistically significant (*P* = 0.078). SLE, systemic lupus erythematosus; CNS, central nervous system; NPSLE, neuropsychiatric lupus

Patients in the CNS infections group were followed up for 47 ± 57 months, but 18 patients (19%) were lost to follow-up. Similarly, 19 patients (20%) in the NPSLE group were lost to follow-up. Thirty patients (31.6%) in the CNS infections group died during the follow-up, and 26 patients (86.7%) died within the first year due to respiratory failure or cerebral hernia. Fourteen patients (14.7%) in the NPSLE group died. Both the total and 1-year mortality rates of the CNS infections group were significantly higher than those of the NPSLE group (total mortality 31.6% vs. 14.7%, 1-year mortality 27.4% vs. 12.6%, both *P* < 0.05) (Fig. 2a).

The prevalence rate of CNS infections with SLE in our in-patient center with 5-year interval frame was 1.4% ± 1.4%, while the rate of NPSLE was 17.6% ± 4.6% (Additional file 2). And the prevalence of CNS infections did not drastically change across this time frame, although there was an increase in the prevalence of NPSLE, from 13.1 to 21.4% during this time interval. The change of etiology for CNS infection in this cohort is shown in Additional file 3, where we can see that Gram-positive bacteria and *Cryptococcus* were the predominant pathogens throughout the whole time period.

### Diverse characteristics and prognosis in different CNS infections

We categorized CNS infection patients into three subgroups according to their etiological findings as follows: bacterial (*n* = 27), cryptococcal (*n* = 18), and mycobacterial (*n* = 11) infections. Patients with bacterial or mycobacterial infections tended to manifest more consciousness disturbance (59.3%, 54.5% vs. 16.7%, *P* < 0.05) and meningeal irritation signs (81.5%, 72.7% vs. 38.9%, *P* < 0.05), compared to cryptococcal infections (Additional file 4). Nearly half of the total deaths (13/30, 43.3%) occurred in the bacterial infection subgroup. The first year mortality rate in the bacterial subgroup was higher than the other two subgroups (37.0% vs. 16.7% and 27.3%). In the survival analysis, the bacterial subgroup showed the worst outcome among the three subgroups (Fig. 2b).

### Characteristics and prognosis of key points for the differential diagnosis of CNS infections from NPSLE

In the multivariate logistic regression analysis, compared with the NPSLE group, the CNS infections group had a longer disease duration (21.0 [3.0–50.0] vs.1.0 [0–22.0] months, OR = 5.2, 95%CI 1.1–24.5, *P* < 0.05), more frequent pyrexia (96.8% vs. 23.2%, OR = 34.3, 95%CI 5.2–226.7, *P* < 0.001), and PMN leukocytosis in the CSF (45.6% vs. 0.5%, OR = 1.09, 95%CI 1.00–1.19, *P* < 0.05) but significantly decreased CSF glucose (2.0 ± 1.3 vs. 3.3 ± 0.9 mmol/L, OR = 13.7, 95%CI 2.1–85.8, *P* < 0.01). Together, these results may be used as clues to distinguish CNS infections from NPSLE. Conversely, the existence of hypocomplementemia seemed to be a strong suggestive index of NPSLE over CNS infections (44.6% vs. 77.4%, OR = 0.08, 95%CI 0.02–0.41, *P* < 0.01) (Table 2).

**Table 2:** A multivariate logistic regression analysis of key points for discriminating CNS infections from NPSLE

Variables	*P* value	OR	95%CI
SLE disease duration^&^ ≥ 12 months	**< 0.05**	5.2	1.1–24.5
Pulse GCs*	0.070	7.7	0.8–70.7
Fever^§^	**< 0.001**	34.3	5.2–226.7
CSF glucose ≤ 2.2 mmol/L^§^	**< 0.01**	13.7	2.1–85.8
CSF PMN leukocytosis^§^	**< 0.05**	1.10	1.00–1.19
Hypocomplementemia^§^	**< 0.01**	0.08	0.02–0.41

*CNS* central nervous system, *NPSLE* neuropsychiatric lupus erythematosus, *GCs* glucocorticoids, *CSF* cerebrospinal fluid, *PMN* polymorphonuclear leukocyte

^&^Disease duration from SLE diagnosis to CNS infections or NPSLE onset

*Evaluated medical history before the diagnosis of CNS infection or NPSLE

^§^Evaluated within 2 weeks of CNS infection or NPSLE onset

Significant *p* values are shown in bold typeface

### Establishment of a simplified scoring system for discriminating CNS infections from NPSLE in SLE patients

Through a univariate analysis and multivariate logistic stepwise regression, 4 items consisting of longer disease duration, fever, CSF, PMN ratio, significantly decreased CSF glucose, and absence of hypocomplementemia were established as the vital risk factors for discriminating CNS infections from NPSLE in SLE patients. CSF examinations, measuring intracranial pressure, WBC count, and protein levels, were also included for their crucial clinical significance evaluated by the MDT. As such, the above 8 items were integrated to establish a simplified scoring system, SSS-8, to assist the rapid recognition of CNS infection in SLE patients. Then, 75 out of 95 cases in the CNS infections group and NPSLE group were randomly selected and combined into one group, the establishment group, and the cutoff value for each item was decided via ROC. The remaining 20 cases were assigned as the verification group (Table 3).

**Table 3:** Simplified scoring system for distinguishing CNS infections from NPSLE

Item	Score
Disease duration^&^ ≥ 12 months	1
Fever^§^	1
Intracranial pressure ≥ 300 mmH_2_O^§^	1
WBCs in CSF ≥ 20/μL^§^	1
PMN ratio in CSF ≥ 0.5%^§^	1
Protein level in CSF ≥ 0.905 g/L^§^	1
Glucose level in CSF ≤ 2.2 mmol/L^§^	1
Absence of hypocomplementemia	1
Total	8

*CNS* central nervous system, *NPSLE* neuropsychiatric lupus erythematosus, *CSF* cerebrospinal fluid, *PMN* polymorphonuclear leukocyte

^&^Disease duration from SLE diagnosis to CNS infections or NPSLE onset

^§^Evaluated within 2 weeks of CNS infection or NPSLE onset

**Fig. 3:**
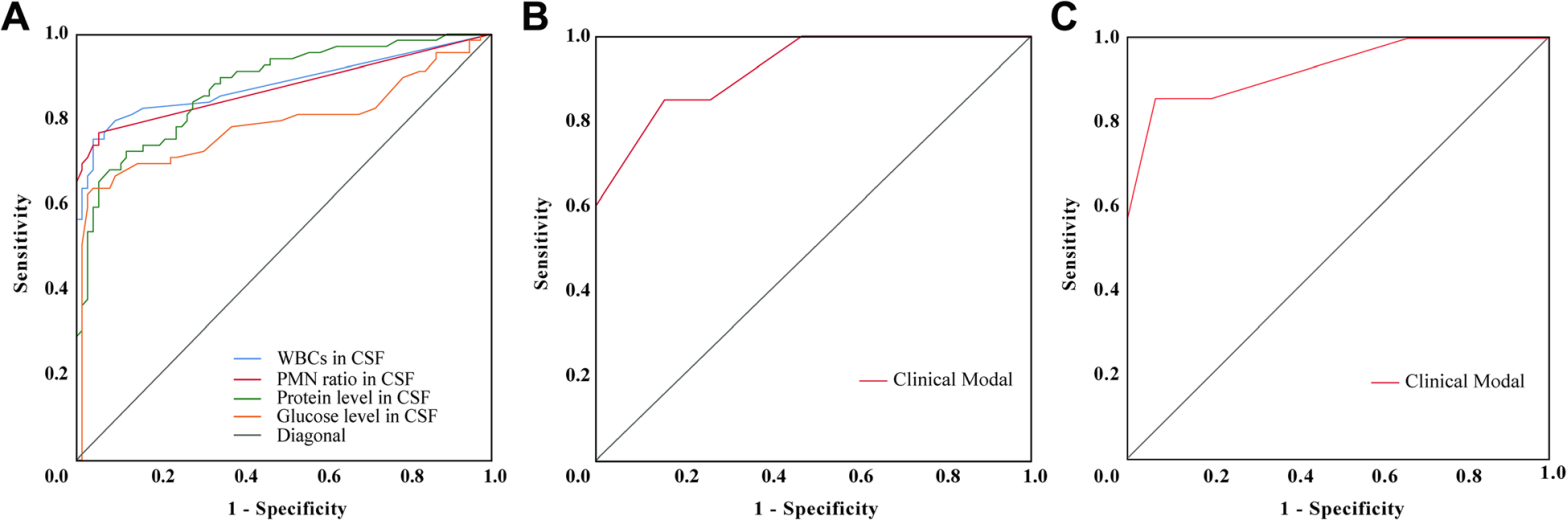
ROC curve for WBC, PMN, glucose, protein in CSF, and SSS-8. **a** ROC curve for WBC, PMN, glucose, and protein in CSF with area under the curve (AUC) being 0.88 (95%CI 0.82–0.94), 0.88 (95%CI 0.81–0.94), 0.88(95%CI 0.82–0.93), and 0.80 (95%CI 0.73–0.88), respectively. The cutoff values for these four indexes are 20/μL, 0.5%, 0.905 g/L, and 2.2 mmol/L, respectively. **b** ROC curve for simplified scoring system with 8 items (SSS-8). AUC is 0.93 (95%CI 0.86–1.00). The cutoff value for distinguishing CNS infections and NPSLE is 4 with a sensitivity of 85.0% and a specificity of 85.0%. **c** ROC curve for verifying SSS-8 in newly onset suspected CNS infection and NPSLE cases. The AUC is 0.93 (95%CI 0.80–1.00). The sensitivity and specificity are 85.7% and 93.3%, respectively. ROC, receiver operating characteristic; WBC, white blood cell; CSF, cerebrospinal fluid; AUC, area under the curve; NPSLE, neuropsychiatric lupus erythematosus; PMN, polymorphonuclear leukocyte

The cutoff value of WBC in CSF, PMN ratio, CSF protein, and CSF glucose level in CSF were 20/μL, 0.5%, 0.905 g/L, and 2.2 mmol/L, respectively (Fig. 3a) with the AUC being 0.88 (95%CI 0.82–0.94), 0.88 (95%CI 0.81–0.94), 0.88(95%CI 0.82–0.93), and 0.80 (95%CI 0.73–0.88), respectively. Notably, nearly 93% of patients in the cohort had normal blood glucose levels. An ICP over 300 mmH_2_O and disease duration longer than 1 year were also considered significant (Tables 1 and 2). Thus, in SSS-8, the presence of each item was assigned to 1 point, and the highest score in SSS-8 was 8 points (Table 3). A score equal to or above 4 points was indicative of a CNS infection with a sensitivity of 85% and specificity of 84.2% (Fig. 3).

### Verification of SSS-8 in SLE patients

Verification of the SSS-8 was first done in the leftover retrospectively collected cases, referred to as the verification group (*n* = 20). With the cutoff value of 4, a sensitivity of 85% and a specificity of 85.0% with the AUC being 0.93 (95%CI 0.86–1.00) can be obtained for discriminating CNS infections from NPSLE (Fig. 3b).

We then verified SSS-8 in a group of SLE patients (*n* = 22) with a recent onset of neuropsychiatric symptoms with suspected CNS infection or NPSLE, who were admitted to PUMCH after January 2018. Patients in this cohort were scored using the SSS-8, and possible diagnosis was predicted according to SSS-8. Their final clinical diagnosis and etiology findings were traced and compared with the SSS-8 prejudgment. In brief, seven patients were confirmed to be definitive CNS infection with etiology evidence obtained later; detailed information is exhibited in Table 4. AUC for SSS-8 in this cohort was 0.93 (95%CI 0.80–1.00). The sensitivity and the specificity are 85.7% and 93.3%, respectively. Positive predictive value and negative predictive value are 85.7% and 93.3%, respectively (Fig. 3c).

**Table 4:** Baseline demographics, CNS features, and SSS-8 of seven patients diagnosed with CNS infections

Case	Gender	Age at SLE diagnosis (years)	Disease duration (months)	Neuropsychiatric symptoms	CSF examination					Hypocomplementemia	SSS-8	Possible CNS infections	Final diagnosis	Etiology	Concordance of prejudge with final diagnosis
					Pressure (mmH_2_O)	WBC (/μL)	PMN ratio (%)	Protein (g/L)	Glucose (mmol/L)						
1	F	35	1	Fever, headache, hemiplegia	310	580	94	1.07	4.23	No	6	Yes	Brain abscess	*Listeria monocytogenes*	Yes
2	F	37	2	Fever, headache, hemiplegia	260	59	1	0.6	2.8	No	4	Yes	Brain abscess	Unknown	Yes
3	F	54	5	Fever, cognitive dysfunction, consciousness disturbance	135	690	92.4	8.83	0.9	Yes	5	Yes	Meningoencephalitis	Unknown	Yes
4	F	53	1	Fever, headache, vomiting, consciousness disturbance	> 330	269	4.9	2.42	2.7	Yes	5	Yes	Meningitis	*Mycobacterium*	Yes
5	F	12	3	Fever, headache	160	50	12	1.5	2.0	Yes	5	Yes	Meningitis	*Fusarium*	Yes
6	F	12	122	Fever, headache, vomiting	120	1054	98	2.78	0.1	No	7	Yes	Meningitis	Gram-negative *bacillus*	Yes
7	M	26	60	Fever, headache, vomiting, consciousness disturbance	230	6	0	0.45	2.7	Yes	2	No	Brain abscess	*Listeria monocytogenes*	No

*CNS* central nervous system, *SLE* systemic lupus erythematosus, *NPSLE* neuropsychiatric lupus erythematosus, *CSF* cerebrospinal fluid, *WBCs* white blood cells, *PMN* polymorphonuclear leukocyte, *SSS-8* simplified scoring system with 8 items

## Discussion

CNS infections are rare but a life-threatening complication of SLE. The incidence of CNS infections ranges from 0.54 to 2.26% according to the limited available reports [11–13]. In this study, based on a large cohort of patients from the tertiary referral center in China, we identified the incidence of CNS infections among SLE patients was 1.12%. However, it should be noted that the investigation was conducted among in-patients in a single center without including out-patient data, due to the constraint of data integrity, and this may cause slight bias and limit the representation of the true incidence among the whole body of lupus patients in China. One strength of this study is that it is the first of its kind to use a sizable population cohort of more than 8000 SLE patients with comprehensive medical documents and reliable etiological evidence, all of which were carefully reviewed by a MDT. With the large sample size and direct comparison to symptoms of patients with NPSLE, this study highlights the general characteristics and outcomes of CNS infections among SLE patients and provides pivotal risk factors for differentiating CNS infections from NPSLE.

The pathophysiological factors that contribute to CNS infections in SLE patients are complicated and multifactorial. Vigorous treatment with immunosuppressants for controlling lupus disease may be one of the major causes of the susceptibility [8, 9, 14, 15]. Our study reveals that 36.8% of SLE patients with CNS infections had been previously treated with pulse GCs, and roughly 50% of patients were treated with a powerful immunosuppressant like CTX and or MMF, within a year prior to CNS infections (in patients with NPSLE these are 7.4% and 17%, respectively). The average daily dose of prednisone given to patients in the last 6 months of the study was 43.5 mg/day in the CNS infections group, double the dosage given to patients in the NPSLE group, which was 21.8 mg/day. 70.5% patients in the CNS infections group had received at least one DMARD in the past 6 months, compared to 37.9% in the NPSLE group. Furthermore, defects in immune defense and surveillance in SLE may contribute to the susceptibility of infections and malignancy [16–18]. Our study finds that a small proportion of patients (5, 5.3%) had CNS infections at the onset of SLE, before they were given immunosuppressive treatment. On the other hand, infections may trigger a lupus flare by stimulating and activating the immune system. Evidence from the literature suggests that bacterial and viral infections might be involved in the induction, exacerbation, and/or flare of SLE [16, 18, 19] by contributing to an aberrant immune response, predisposing to a tolerance breakdown toward native proteins [19]. Viruses such as EBV, cytomegalovirus, and Parvovirus B19 are frequently reported as environmental triggers in SLE autoimmunity [20–22], which may partly explain the collateral active lupus disease with bacterial/virus infection in our study.

In our cohort, *Cryptococcus* was the most common pathogen identified in SLE patients with CNS infections, followed by mycobacteria and *Listeria monocytogenes*, which is consistent with the previous reports [11, 13]. Comparisons among these three categories of pathogens revealed that patients infected with *Cryptococcus* relatively had a better outcome (Fig. 2b), probably due to a chronic inclination in the disease course. Eleven patients had CNS infections with *Mycobacterium* confirmed by positive acid-fast bacilli (six patients), high-copy TB DNA (two patients), and culture (three patients). *Mycobacterium tuberculosis* is a type of intracellular bacteria. Impaired cell-mediated immunity, especially defects in the macrophages, renders susceptibility in SLE patients [23]. *L. monocytogenes* was the major pathogen identified in the bacterial infection subgroup. Patients with Gram-negative bacteria infection had the worst outcome, with a mortality rate of 83.3%. Fifty percent of them died within 10 days after diagnosis, and the remaining 33.3% of patients died within 1 month.

Identifying biomarkers to assist the prompt diagnosis of infections and distinguish infections from a SLE flare is essential for preventing poor outcomes [9, 17, 19, 24, 25]. The anti-dsDNA antibody titer and the presence of hypocomplementemia have been proposed as biomarkers for lupus [26], as well as CD 64-Fc receptor expression [21, 27]. Other studies, looking at the prognostic factors of infections and SLE flares [8, 14] suggests that the administration of CTX and intravenous corticosteroids increase the risk of infections. However, when it comes to CNS infections, there may be other factors that are leading to the onset of infections. Our study reveals that besides a higher dose of daily corticosteroid treatment, and previous vigorous DMARD usage, patients who have a longer disease duration (≥ 1 year), and patients with a previous history of herpes zoster infection, have a higher risk of CNS infections. This underscores the need for more intense monitoring of the primary disease during treatment and meticulous balance of the intensity of lupus treatment to minimize the risk of severe infection. Patients with neuropsychiatric symptoms, concurrent fever, persistent and violent headache, and consciousness disturbances, other than seizure disorders and cognitive dysfunction, should always be scrutinized for the possibility of CNS infections. Our data shows that CSF examination is extremely important. CSF test with extremely high intracranial pressure, leukocytosis predominant with PMN, and increased protein but decreased glucose levels is indicative of CNS infections instead of NPSLE.

We also aimed to construct a simplified and practical evaluation system to help prompt differentiation between these 2 severe and confusing complications, for doctors treating patients with SLE. By integrating 79% SLE patients with CNS infections (*n* = 75) and NPSLE (n = 75) via multivariate regression and ROC analysis, as well as suggestions made by a multiple disciplinary team, we generated an 8-item simplified scoring system, called SSS-8. The 8 items were a long disease duration (≥ 1 year), fever (> 37.3 °C), absence of hypocomplementemia and super high intracranial pressure (≥ 300 mmH_2_O), CSF leukocytosis (WBC ≥ 20/μL), PMN predominance in CSF (≥ 0.5%), increased protein level (≥ 0.905 g/L), and/or decreased glucose level (≤ 2.2 mmol/L) in CSF. The presence of each item was assigned 1 point, and patients who scored 4 points or above were considered as more inclined to CNS infections. This scoring system was first verified in the remaining 20 cases of the retrospective SLE cohort and obtained an AUC of 0.93 (95%CI 0.86–1.00) with a sensitivity and specificity for modal of 85.0% and 85.0%, respectively. We then further verified the SSS-8 in a small group of SLE patients with a recent onset of neuropsychiatric symptoms and undetermined diagnosis of CNS infection or NPSLE. Their final clinical diagnosis was recorded and compared to their predicted diagnosis using the SSS-8 scale. Our SSS-8 scale evaluation predicted 6/7 patients had CNS infections, and these cases were confirmed with etiologic evidence and/or clinical decision. Further, the SSS-8 predicted 14/15 patients had non-CNS infections and turned out to have the final clinical diagnosis of NPSLE. However, team with limited number of experts, this scoring system does have some shortcomings: it is generated from a limited sample, and each case was discussed among a multidisciplinary team. Also, CSF lab tests are not sensitive for brain abscess which is not uncommon in CNS infections in SLE. Further verification in a large prospective cohort and optimization with better predictive markers or cutoff values are necessary before the widespread use of this rubric.

Primary NPSLE events, which account for 1/3 of cases with neuropsychiatric manifestation in major SLE cohorts, are a consequence of either inflammatory mediators and autoantibodies, or thrombosis and microvasculopathy. The remaining 2/3 of cases with neuropsychiatric manifestations are due to the secondary causes like treatment, infections, and metabolic abnormalities [28, 29]. To establish the diagnosis of NPSLE, causes other than SLE that might be responsible for the neuropsychiatric symptoms need to be excluded. In this study, we relied on ACR 1999 criteria and specialists’ opinions for NPSLE classification and enrollment. And all NPSLE patients had received contemporary routine screening tests to rule out infections, for these patients might need to be treated promptly with high-dose or even pulse GC treatment. Case files were reviewed by two rheumatologists; also, a specialist of infectious disease was asked to join in for further exclusion of infection possibilities. We agree that although ACR1999 criteria were a milestone in the classification and nomenclature of NPSLE, limitations exist and confounders still remain [28]. In the future, the presence of NPSLE-associated autoantibodies, or cytokines in serum/CSF, combined with MRI findings may add to the diagnosis criteria and contribute to a more accurate classification of NPSLE from other confounding illnesses.

## Conclusions

Discrimination between central nervous system infections and SLE flares is extremely challenging in medical clinics. The proposed scoring system, SSS-8, which examines the disease duration, presence of fever, and absence of hypocomplementemia, together with CSF analysis showing extremely high intracranial pressure, high WBC levels predominant with PMN leukocyte, and high protein levels with a decrease in glucose levels, may help clinicians to promptly, and more adequately, distinguish CNS infections from NPSLE.

## Additional files

Additional file 1: Flowchart of screening of SLE patients with CNS infections and NPSLE. (PDF 91 kb)

Additional file 2: The prevalence rate of CNS infections with SLE vs. NPSLE. (PDF 278 kb)

Additional file 3: The change of etiology of 59 SLE patients with CNS infections with a 5-year interval frame. (PDF 350 kb)

Additional file 4: CNS infections in SLE patients with different etiology findings. (PDF 66 kb)

## Data Availability

The dataset used and/or analyzed during the current study is available from the corresponding authors on reasonable request.

## Abbreviations

ACR: American College of Rheumatology
AUC: Area under the curve
CALAS: Cryptococcal Antigen Latex Agglutination System
CNS: Central nervous system
CSF: Cerebrospinal fluid
CT: Computed tomography
CTX: Cyclophosphamide
DMARDs: Disease-modifying antirheumatic drugs
EBV: Epstein-Barr virus
GCs: Glucocorticoids
MDT: Multiple disciplinary team
MMF: Mycophenolate mofetil
NPSLE: Neuropsychiatric lupus erythematosus
PCR: Polymerase chain reaction
PMN: Polymorphonuclear leukocyte
PNS: Peripheral nervous system
PUMCH: Peking Union Medical College Hospital
ROC: Receiver operating characteristic
SLE: Systemic lupus erythematosus
SLEDAI-2K: Systemic lupus erythematosus disease activity index 2000
SSS-8: Simplified scoring system with 8 items
WBC: White blood cell
