# Supplementary material for: Clinical features of central nervous system infections and experience in differential diagnosis from neuropsychiatric lupus erythematosus in a cohort of 8491 patients with systemic lupus erythematosus
Source: Arthritis Res Ther. 2019 Aug 19;21:189. doi: 10.1186/s13075-019-1971-2 (PMC6701089; doi:10.1186/s13075-019-1971-2)
Supplement: Supplementary file 1 — Flowchart of screening of SLE patients with CNS infections and NPSLE. (PDF 91 kb) [file 13075_2019_1971_MOESM1_ESM.pdf]

Supplementary file 1. Flowchart of screening of SLE patients with CNS infections and NPSLE

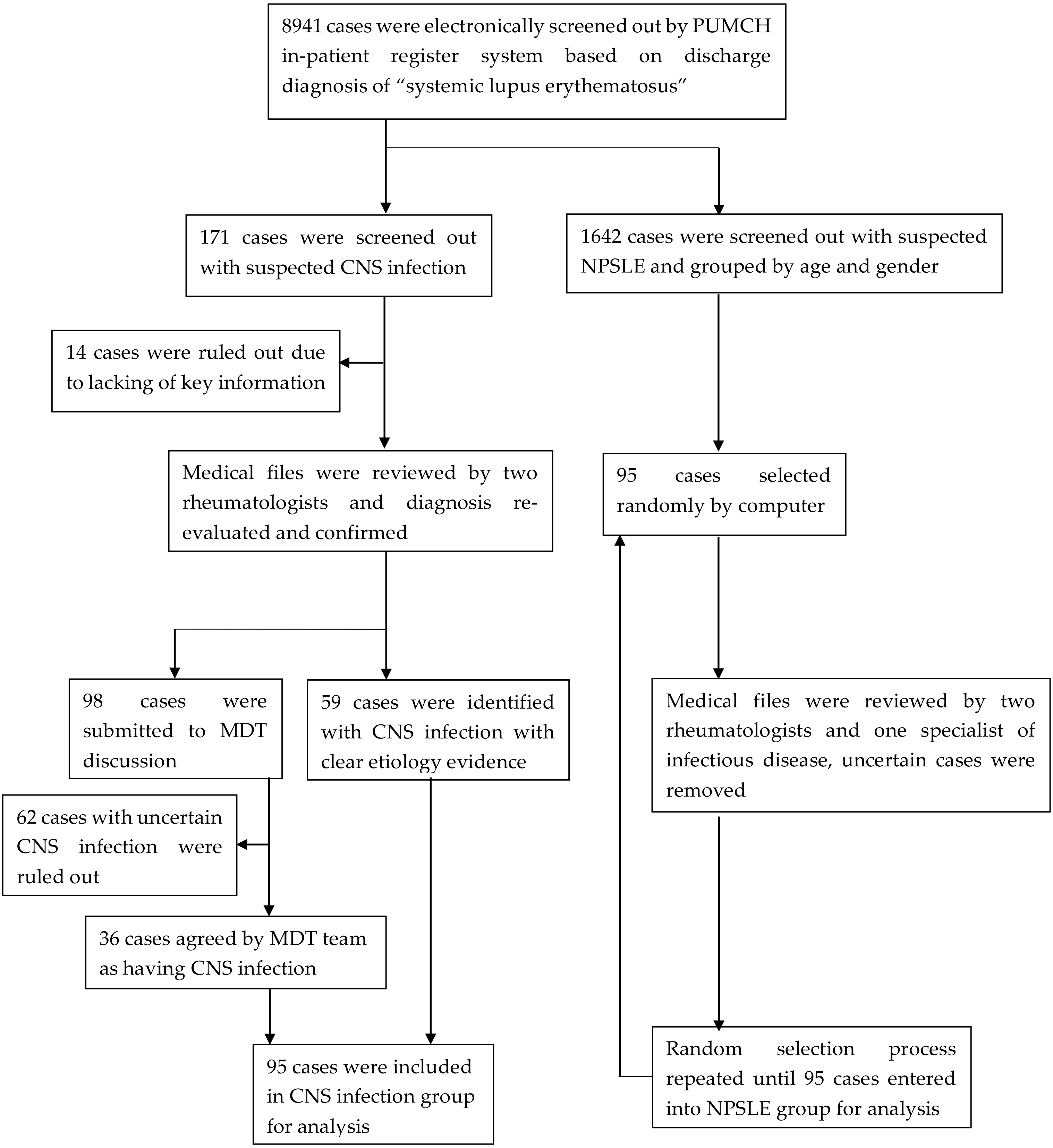

PUMCH: Peking Union Medical College Hospital; CNS: central nervous system; NPSLE: neuropsychiatric lupus erythematosus; MDT: multiple disciplinary team
