# Supplementary material for: Clinical features of central nervous system infections and experience in differential diagnosis from neuropsychiatric lupus erythematosus in a cohort of 8491 patients with systemic lupus erythematosus
Source: Arthritis Res Ther. 2019 Aug 19;21:189. doi: 10.1186/s13075-019-1971-2 (PMC6701089; doi:10.1186/s13075-019-1971-2)
Supplement: Supplementary file 3 — The change of etiology of 59 SLE patients with CNS infections with a 5-year interval frame. (PDF 350 kb) [file 13075_2019_1971_MOESM3_ESM.pdf]

Supplementary file 3. The change of etiology of 59 SLE patients with CNS infections with a 5-year interval frame

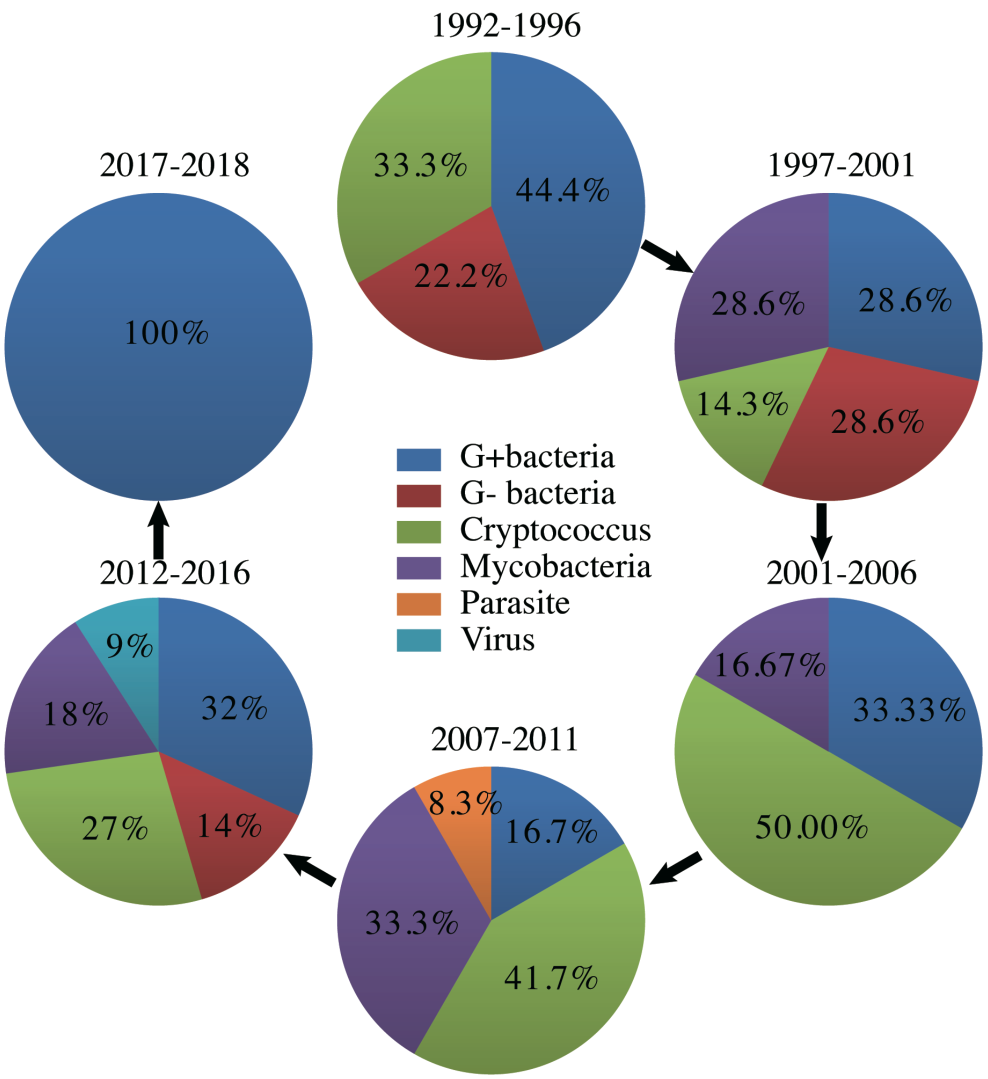

G+ bacteria: gram-staining positive bacteria; G- bacteria: gram-staining negative bacteria;
