# Supplementary material for: Clinical features of central nervous system infections and experience in differential diagnosis from neuropsychiatric lupus erythematosus in a cohort of 8491 patients with systemic lupus erythematosus
Source: Arthritis Res Ther. 2019 Aug 19;21:189. doi: 10.1186/s13075-019-1971-2 (PMC6701089; doi:10.1186/s13075-019-1971-2)
Supplement: Supplementary file 4 — CNS infections in SLE patients with different etiology findings. (PDF 66 kb) [file 13075_2019_1971_MOESM4_ESM.pdf]

Supplementary file 4. CNS infections in SLE patients with different etiology findings

	Bacterium (n=27)	Cryptococcus (n=18)	Mycobacterium (n=11)	P value
Interval from CNS onset to CNS infections diagnosis, days, median (IQR)	4 (2-14)	22 (6-51)	24 (7-55)	**<0.01**
SLEDAI-2K ≥10 at infections, n (%)	13 (48.1)	3 (16.7)	1 (9.1)	**<0.05**
Treatment, n (%)				
Pulse GCs	7 (25.9)	9 (50.0)	4 (36.4)	0.256
CTX/MMF in past 1 year	13 (48.1)	11(61.1)	8(72.7)	0.350
CNS manifestation, n (%)				
Consciousness disturbance	16 (59.3)	3 (16.7)	6 (54.5)	**<0.05**
Meningeal irritation	22 (81.5)	7 (38.9)	8 (72.7)	**<0.05**
CSF examination				
Pressure >300 mmH_2O, n (%)	12 (44.4)	12 (66.7)	7 (63.6)	0.347
Protein, g/L, mean (SD)	1.65 (1.07)	1.50 (1.41)	1.73 (0.80)	0.859
Glucose, mmol/L, mean (SD)	1.77 (1.17)	2.01 (1.61)	1.71 (1.21)	0.787
Laboratory data				
Peripheral WBCs, 10^6/L, mean (SD)	11192 (8295)	7893 (4031)	7966 (3345)	0.172
ESR, mm/h, mean (SD)	62.5 (43.5)	52.3 (43.4)	40.6 (28.2)	0.320
Mortality rate#, n (%)	10 (37.0)	3 (16.7)	3 (27.3)	0.221

SLE: systemic lupus erythematosus; CNS: central nervous system; SLEDAI-2K: systemic lupus erythematosus disease activity index 2000; GCs: glucocorticosteroids; CTX: cyclophosphamide; MMF: mycophenolate mofetil; CSF: cerebrospinal fluid; WBCs: white blood cells; ESR: erythrocyte sedimentation rate; # Evaluated at one year of the diagnosis of CNS infections or NPSLE.
